# Stabilized HIV-1 envelope glycoprotein trimers for vaccine use

**DOI:** 10.1097/COH.0000000000000363

**Published:** 2017-02-21

**Authors:** Max Medina-Ramírez, Rogier W. Sanders, Quentin J. Sattentau

**Affiliations:** a Department of Medical Microbiology, Academic Medical Center, University of Amsterdam, Amsterdam, The Netherlands; b Department of Microbiology and Immunology, Weill Medical College of Cornell University, New York, USA; c Sir William Dunn School of Pathology, University of Oxford, South Parks Road, Oxford, UK

**Keywords:** broadly neutralizing antibodies, chemical cross-linking, HIV-1 envelope glycoproteins, mutagenesis, soluble trimers, stabilization, trimer structure

## Abstract

**Purpose of review:**

To provide an update on the latest developments in the field of HIV-1 antibody-based soluble envelope glycoprotein (Env) trimer design for vaccine use.

**Recent findings:**

The development of soluble native-like HIV-1 Env trimer immunogens has moved the field of antibody-based vaccine design forward dramatically over the past few years with refinement of various stabilizing approaches. However, despite this progress, significant challenges remain. Firstly, although trimers are relatively stable in solution, they nevertheless sample different conformational states, some of which may be less relevant to binding and induction of broadly neutralizing antibodies (bNAbs). Secondly, these trimers expose unwanted immunodominant surfaces that may distract the adaptive immune response from recognizing more immunorecessive but conserved neutralization-relevant surfaces on the trimer. The availability of atomic-resolution structural information has allowed guided design of mutations that have further stabilized trimers and allowed reduced exposure of unwanted epitopes. Moreover, chemical cross-linking approaches that do not require structural information have also contributed to trimer stabilization and selection of particular conformational forms. However, current knowledge suggests that strategies additional to trimer stabilization will be required to elicit bNAb, including targeting naïve B cell receptors with specific immunogens, and guiding B cell lineages toward recognizing conserved surfaces on Env with high affinity.

**Summary:**

This review will give a perspective on these challenges, and summarize current approaches to overcoming them with the aim of developing immunogens to elicit bNAb responses in humans by active vaccination.

## INTRODUCTION

An effective HIV-1 vaccine is expected to require the induction of protective antibodies, most probably neutralizing antibodies (NAbs). The concept that NAbs might form the basis for a protective vaccine against HIV-1 infection was first tested in nonhuman primates (NHPs), and revealed that gp120-based immunogens elicited antibody responses that neutralized homologous virus and protected NHPs from challenge [1]. However, initial optimism was subdued with the realization that viruses used to prepare the immunogens and carry out the neutralization and challenge studies were neutralization sensitive as a result of multiple cell culture passages [2,3]. Such viruses are now termed Tier-1.

Subsequently, the field moved to so-called “primary isolates” - viruses isolated and with limited passage in primary cells - as they represented more closely the neutralization phenotype typical of clinical isolates (termed Tier-2 viruses) [4–6]. In parallel came realization that antibodies elicited by immunization with gp120 only weakly engaged gp120 on the surface of virions or infected cells in the context of the functional viral envelope glycoprotein (Env) trimer [7], whereas, patient sera that neutralized primary isolates bound cell surface functional Env more avidly [8]. These results engendered the straightforward concept that antibody binding to the functional Env trimer is necessary and sufficient for neutralization [9–11]. Neutralization by tight binding (i.e., high affinity and/or avidity interactions) anywhere on the functional Env trimer made straightforward mechanistic sense, because engaging gp120 with an IgG molecule was predicted to prevent virus attachment and entry via steric or conformational inhibition of receptor engagement [11–13], and ligating gp41 would most likely inhibit viral fusion. Although some modifications to the basic hypothesis have been proposed, based for example on the observation that some NAb specificities irreversibly inactivate Env by inducing trimer destabilization and/or dissociation [14–17], or that introduce allosteric changes that alter receptor binding [18], the basic tenet of neutralization = functional trimer binding has largely held true. The importance for neutralization of trimer occupancy by antibody determined by affinity has been exemplified by bNAb lineage members with rapid off-rates that fail to neutralize [19], and by antibody avidity in experiments showing that appropriately spaced linkers allow intra-trimer cross-linking on virions that enhance neutralization potency [20].

The idea that the functional Env trimer was the most relevant target for NAb drove the production of its soluble form. However, because Env is made up of noncovalently linked gp120-gp41 heterodimers, expression without a membrane context resulted in structural instability in solution. The next step therefore was to stabilize these trimers, and first generations of stable soluble trimers were engineered to eliminate the gp120-gp41 cleavage site to generate gp140 constructs, in some cases fused to extrinsic trimerization domains [21,22]. Multiple gp140 antigens were tested for antigenicity, immunogenicity, ability to elicit NAbs and protection from challenge in NHPs. However, despite reports of modest improvements in immunogenicity over soluble gp120 [23], these types of trimer consistently failed to elicit bNAbs or even autologous Tier-2 NAbs [24–26,27^▪^].

Why was this? Fast forward to the present day, and we know from antigenic, biochemical, and electron microscopic morphological analyses that these trimers did not mimic the structure of the functional trimer on the virus, and therefore could not in principle fulfill the criterion of eliciting antibodies that would bind to it [28–30]. Indeed these first generation trimers (or pseudotrimers [31]), were much like individual gp120 molecules linked to a postfusion gp41 molecule, and so the only potential advantage over soluble gp120 might have been multivalency of B cell receptor (BCR) engagement with ensuing enhanced B cell activation. This might explain the reports of modestly enhanced NAb elicitation observed with some of these reagents [24,32].

### bNAbs, monomeric gp120 and trimeric Env

Our current knowledge of the epitope specificity of bNAbs isolated from infected individuals invokes at least a partial explanation for why gp120 monomers and postfusion gp41 trimers fail to elicit bNAbs. Three of the six known bNAb epitope clusters are either strongly quaternary fold-dependent (the trimer apex and the gp120-gp41 interface surfaces [33]), or partially quaternary-dependent (the CD4 binding site, CD4bs [34]). Thus, use of monomeric gp120 or gp140 pseudotrimers will fail to generate these bNAbs. However, the failure of gp120 and incorrectly-folded gp140 trimers to elicit Tier-2 NAbs can not only be explained by lack of correct trimerization, as the epitopes of other bNAbs including the N332 supersite and some CD4bs bNAbs are largely maintained [33].

Env molecular evasion strategies that may account for the failure of gp120 to elicit N332 bNAbs include the necessity to engage not only protein surface, but also glycan moieties. Since Env glycans are host cell derived, they are self and therefore tolerated, or at least poorly immunogenic for B cell recognition [35–37]. This idea is reinforced by the observation that many glycopeptide-reactive bNAbs have unusual structural features including long CDRH3 loops and have undergone extensive somatic hypermutation [19,38–40], suggesting that BCR triggering by such surfaces is a rare event. Another concern relates to the focusing of helper T and B cell responses toward non-neutralizing immunodominant surfaces, principal amongst which is the hypervariable gp120 V3 loop, which is not a bNAb target and is highly plastic, allowing mutations to evade recognition [41,42].

That the CD4bs is largely conserved on monomeric gp120 is clear from binding analyses showing high-affinity engagement. However, more recent structures of trimeric Env reveal that many CD4bs-specific bNAbs engage a second gp120 protomer within the trimer [34]. Thus although gp120-based immunogens, including engineered outer domain constructs such as eOD [43], can elicit antibodies that bind gp120 and engage VRC01-class naïve human B cells [44^▪^] or their transgenic counterparts in humanized mice [45^▪^], they so far fail to show bNAb activity. A related and similarly difficult challenge is the ‘angle of approach’ of the BCR to the CD4bs embedded within the trimer. The CD4bs on each gp120 protomer is located such that this surface can only be engaged at a very specific vectorial angle [46–48]. A further hurdle to BCR recognition of the CD4bs is the proximity of glycans [49,50] that may limit receptor on-rate to a level that makes successful engagement a rare event.

**Box 1:**
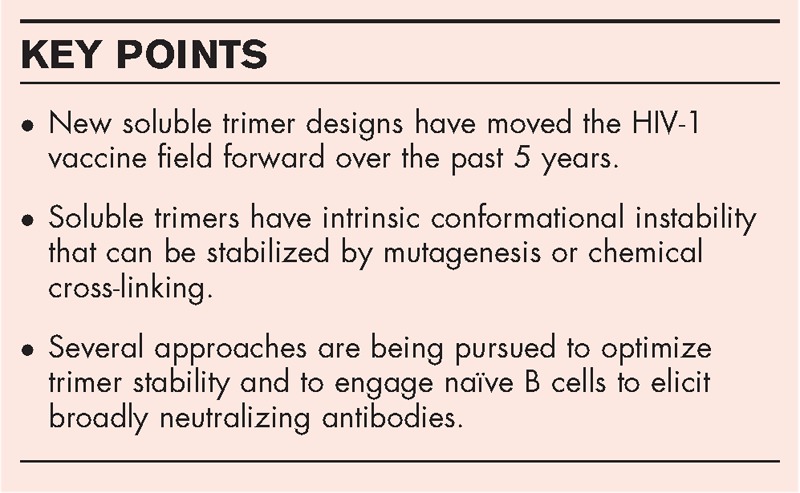
no caption available

### Stable native-like trimers

As an alternative stabilization approach to cleavage-site removal in the aforementioned first-generation pseudotrimers, researchers stabilized the gp120-gp41 interactions with an intermolecular disulfide bond (SOS gp140), supplemented with an I559P substitution to improve trimerization (SOSIP gp140) [51–53]. Additional modifications and combination with the sequence of BG505 eventually yielded trimers that were considered “native-like” as assessed by electron microscopy (EM) [54–56]. Furthermore, these trimers are recognized by most bNAbs tested, except those binding the truncated MPER domain. Overall, the antigenicity of the BG505 SOSIP.664 trimer closely reflects that of the native BG505 Env spike, as judged by a strong correlation between the outcomes of trimer-binding and virus-neutralizing assays [27^▪^]. This correlation also forms a strong retrospective argument for the previously invoked hypothesis that trimer antibody occupacy is the major determinant for virus neutralization.

The BG505 SOSIP.664 trimers allowed elucidation of the Env structure in both unligated and bNAb-complexed forms. The first high-resolution structures of the BG505 SOSIP.664 trimer in complex with bNAbs PGT122 targeting the N332 supersite cluster and PGV04 targeting the CD4bs were obtained by x-ray crystallography [57] and cryo-EM [34] respectively. More trimer structures followed including BG505 SOSIP.664 and trimers from other isolates [47,48,58^▪^,^59^,^60^]. The highest resolution structure (3 Å) allows detailed interpretation of the atomic interactions between and within individual protomers [61]. Some concerns remained as to whether the SOSIP substitutions altered trimer structure, but a recent high-resolution cryo-EM structure of full-length, membrane-derived Env lacking SOSIP substitutions revealed that its overall structure is very similar to the soluble BG505 SOSIP.664 trimer [62^▪▪^]. The only exception was the region around residue 559, where the proline substitution in the SOSIP.664 trimer breaks a helix as it was designed to do [63]. These structures have yielded tremendous mechanistic information on how the trimer works as a fusion machine [64]. They have also provided atomic-level information on the nature of bNAb epitopes [34,47,57,59–61,65] and have visualized important glycans involved in antibody binding or evasion [58^▪^]. SOSIP trimers were also instrumental in the identification and characterization of novel bNAbs [19,60,66]. Finally, SOSIP trimer structural analysis facilitated the design of alternative and/or complementary approaches to stabilize gp140 trimers [67–69,70^▪▪^].

Immunogenicity studies in animals using these SOSIP trimers revealed progress towards elicitation of NAbs able to neutralize autologous Tier-2 viruses, and have pointed the way toward next steps in immunogen design. When rabbits were immunized with BG505 SOSIP.664 trimers, they induced strong and consistent NAb responses against the parental Tier-2 primary isolate BG505, something not previously observed with Env-based immunogens. Similarly, SOSIP.664 trimers based on a different clade B isolate (B41) induced strong autologous Tier-2 NAbs in rabbits [27^▪^], suggesting that this may be a somewhat generalizable phenomenon. By contrast, the corresponding BG505 gp120 and gp140 pseudotrimer constructs induced weaker or undetectable NAb responses. NAb responses were also induced by BG505 SOSIP.664 trimers in macaques, although at lower titers than in rabbits [27^▪^]. Although strong heterologous NAb responses against Tier-1 viruses were induced, heterologous NAbs against Tier-2 viruses were sporadically and weakly elicited, a challenge that is guiding future design approaches.

Mapping of the NAb serum responses revealed that the autologous Tier-2 NAb response in the rabbits was dominated by specificities targeting a hole in the glycan shield of BG505 viral Env centered around residues 241 and 289 where most viral Envs have N-linked glycans [71]. McCoy *et al.* [72] isolated autologous Tier-2 neutralizing monoclonal Abs (MAbs) from rabbits immunized with BG505 SOSIP.664. Most of these MAbs indeed targeted the glycan hole around residues 241 and 289. Whether these glycan hole-directed NAb responses can be exploited and broadened remains to be seen. Other NAb specificities were also induced, and are currently undergoing characterization [27^▪^,^71^].

The serological responses induced by BG505 SOSIP.664 trimers in rabbits were dominated by responses against V3 [27^▪^]. Although V3 antibodies neutralize Tier-1 viruses, they usually do not neutralize Tier-2 viruses. A V3-specific MAb was also isolated from BG505 SOSIP.664-immunized rabbits [72], and consistent with the serum responses, this MAb cross-neutralized Tier-1 but not Tier-2 viruses, including the autologous BG505 virus. The SOSIP structures revealed that V3 is largely buried on SOSIP trimers, consistent with the inability of V3 MAbs to neutralize the BG505 virus. However, apparently the V3 loop becomes exposed during immunization and dominates the antibody response as described above. Dampening off-target responses such as those against V3 might be important for further native-like trimer improvement.

### Trimer conformational instability and further stabilization

The concept that the Env trimer might be metastable came from observations that receptor engagement triggered conformational changes required for virus-cell fusion, including V3 loop and gp41 exposure [73]. This led to the paradigm of Env constitutively oscillating between “open” and “closed” states [74–77], with neutralization sensitivity broadly reflecting the dynamics of this process. An equilibrium favoring “open” may allow greater access of otherwise weakly- or non-neutralizing antibodies (non-NAbs) to the trimer surface, whereas “closed” trimer favors bNAb binding [78]. Hydrogen-Deuterium Exchange studies using SOSIP trimers and single molecule FRET studies using virus with fluorescently tagged gp120 molecules to report on changes in inter-protomer proximity directly demonstrated at least 3 distinct Env conformations present both in membrane anchored and soluble Env [48,78–80]. The closed conformation, which corresponded to the lowest energy “ground” state, showed maximum discrimination between bNAb and non-NAb binding, and hence is considered the conformation most relevant to successful immunogen design [48,79,81]. This therefore led to the concept of conformational stabilization of the closed conformation by two approaches: mutagenesis based on structure-guided design, and chemical cross-linking. Structure-based design has been highly successful for eliminating trimer instability and reducing exposure of the V3 loop, and is discussed in detail later. Reducing elicitation of non-NAbs may be important not only for reducing competition for rare bNAb-eliciting B cells as described above, but also for preventing such non-NAb specificities such as V3 loop antibodies from engaging transiently open trimers and locking them into this state, constitutively revealing other non-neutralizing immunodominant epitopes for BCR recognition. Whether these off-target non-NAb responses do indeed interfere with the induction of bNAb responses is currently unclear, but it is certainly reasonable to hypothesize that further stabilization of SOSIP.664 trimers could benefit immunogenicity [77,82].

The atomic-level structures of BG505 SOSIP.664 trimers paved the way for strategies to further stabilize these trimers [48]. Kwon *et al.* reported that introducing a disulfide bond between residues in β3 and β21 reduced the conformational mobility in BG505 SOSIP.664 by fixing the trimer in the unliganded state. This variant, called DS-SOSIP.664, displayed reduced sensitivity to CD4 inducted conformational changes and increased thermostability compared to the parental SOSIP.664 protein [48]. De Taeye *et al.* [77] engineered a set of modifications, including an E64K or H66R change in C1 and an A316W substitution in V3, that collectively reduced conformational flexibility of SOSIP trimers from multiple isolates: BG505 (clades A), B41 and AMC008 (clade B), and ZM197 M (clade C). These stabilized trimers, termed SOSIP.v4 (version 4), showed improved binding of bNAbs while reduced binding of non-NAbs targeting the V3 or the CD4i epitopes, as well as higher thermostability. When used as immunogens in rabbits SOSIP.v4 trimers induced reduced V3-specific responses without impairing autologous Tier-2 NAb responses. More recently, Kong *et al.* [69] redesigned a largely disordered bend in heptad region 1 (HR1) of SOSIP trimers that connects the long, central HR1 helix to the fusion peptide. This modification resulted in a substantial improvement of the yield of well-folded trimers. Finally, Steichen *et al.* [70^▪▪^] used an iterative mammalian-display directed-evolution design approach that yielded BG505 SOSIP.664 derivative trimers with improved yields relative to the parental BG505 SOSIP.664, and that showed reduced binding of V3 non-NAbs.

### Chemical trimer stabilization

Chemical inactivation of viruses had been used for almost a century for inactivating and detoxifying pathogens and their toxins, and has been very successful in multiple human vaccines including polio, influenza, diphtheria, tetanus, and pertussis toxins. Despite the availability of genetic approaches to pathogen attenuation, inactivation, and detoxification, many vaccines still use chemical cross-linking for inactivation and detoxification [83–86]. Protein cross-linking agents not only include aldehydes, principally formaldehyde (FA) but also glutaraldehyde (GLA), and β-propiolactone, a nucleic acid cross-linking agent. Aldehydes preferentially form cross-links with amine groups on lysines, but can also target arginine and other amino acids under certain conditions [87,88]. The formation of cross-links may be deleterious for some antibody epitopes either as a result of global conformational change introduced into the protein by cross-linking of conformationally sensitive regions, or by specifically modifying amino acids within the antibody epitope.

Stabilization of HIV-1 Env by aldehyde treatment was first assessed using infected cells to probe binding of a limited panel of antibodies to membrane anchored-trimers. Results indicated that under gentle cross-linking conditions, antigenicity could be broadly conserved, and reassuringly, binding of a bNAb, IgG1b12, was retained by cross-linking [89]. A subsequent study showed that GLA cross-linking of first generation soluble gp140 trimers reduced the expression of non- and weak-NAbs, focused antibody responses toward the conserved CD4bs, and modestly enhanced neutralization breadth and potency [90]. Aldehyde cross-linking with antibody immunoprecipitation was elegantly harnessed to demonstrate the presence of different conformational states of gp120 and gp140 [91,92]. GLA and other cross-linking agents were subsequently used to stabilize membrane anchored Env prior to detergent extraction and purification for antigenic and immunization analysis [93]. Results obtained were consistent with global preservation of antigenicity, but immunization failed to elicit high titer bNAbs [93].

The development of BG505 SOSIP.664 trimers allowed further development of the cross-linking process. The rationale for chemical cross-linking of these antigens was firstly to introduce additional stabilization, and secondly to allow isolation of specific conformational states of the trimer from the population of trapped “open,” “closed,” and intermediate forms. In Schiffner *et al.* [94], two cross-linkers were tested: GLA and EDC [1-ethyl-3-(3-dimethylaminopropyl)carbodiimide hydrochloride]. EDC is a heterobifunctional zero-length agent that activates carboxyl and amine cross-linking leaving no residual atoms. BNAb binding was minimally perturbed, whereas non- and weak-NAb binding was substantially reduced by cross-linking. The most likely reason for this is that non-NAbs cannot bind tightly to the functional trimer in its “closed” state, and therefore can only bind to those trimers locked into the open state, which form a subgroup of the entire population. Cross-linked trimers were therefore subject to antibody affinity chromatography to either positively select the most favorable antigenic forms, or deselect those with less desirable characteristics. Purification of GLA and EDC-cross-linked trimers using quaternary-specific bNAbs (PGT151 and PGT145) yielded ∼100% well folded trimers (as gauged by negative stain EM), and bound a large panel of bNAbs while they failed to bind, or bound very weakly, non-NAbs [94]. Further selection included depletion of V3 loop reactivity resulting in trimers that essentially failed to bind V3 antibodies [94]. A recent study showed that chemical cross-linking or mutagenesis enhanced stabilization increased stabilization correlated with the induction of NAbs, providing support for pursuing such approaches [95].

### Broadening NAb responses

During natural infection bNAb responses are not a result of encounter with one antigen of fixed composition but are a product of years of co-evolution of virus and B cells. Thus, it seems unlikely that development an HIV-1 vaccine based on a single Env immunogen will be feasible. It is more likely that various Env trimers should be administered in combination or in sequence to guide B cell responses toward neutralization breadth. In this light, four complementary approaches should be explored, that is, empirically composed vaccine regimens; patient-based lineage vaccines; rationally designed germline-targeting approaches; and iteratively designed vaccine regimens.

The first approach involves combination of genetically diverse native-like trimers, such as those based on different clades [71]. The first study that reported on such combinations made use of trimers from the virus strains BG505 (clade A), B41 (clade B), DU422, and CZA97 (both clade C) administered in combination and sequence. Although the study did not yield potent bNAb responses, relevant conclusions could be drawn. First, rabbits developed Tier-2 NAb responses against multiple SOSIP trimers delivered simultaneously or sequentially. Furthermore, clade C SOSIP trimers cross-boosted preexisting NAb responses to the clade A and B trimers. Weak heterologous Tier-2 NAb responses were observed albeit inconsistently and with limited overall breath. It is unlikely that a “magic cocktail” of trimers able to elicit bNAbs exists, but these empirical studies should teach us about shaping B cell responses using combinations of distinct but related antigens.

Empirical immunization experiments ignore the process by which bNAbs develop during natural infection, where cycles of viral escape from NAbs and renewed cycles of somatic hypermutation triggered by viral escape culminates in NAbs that can accommodate enough variation within the respective epitopes to recognize multiple viruses. Patient-based viral Env phylogenetic lineage-based vaccine approaches aim to reproducibly recapitulate such processes [39,96–98]. The basic principle involves sequential immunization with Env proteins identified over time in a patient that developed bNAbs. However, the observations that bNAb development usually takes years and only occurs in a subset of patients, suggests that there are major roadblocks the developmental pathway of bNAbs. Three important factors contribute to the development of bNAbs, that is, viral activation of naïve B cells expressing bNAb-precursors; viral diversification that allows sequential maturation of these B cells; and avoiding negative selection of B cells through poly- or autoreactivity [99]. The selection of patient-derived Env proteins should take these factors into account, in addition to minimizing off-target responses. Recent progress in the development of computational methods that analyze longitudinal viral sequence data provides important information for sequence selection [100].

Rationally designed germline(gl)-targeting approaches rely on the same factors described above for patient-based approaches. The main difference is that the immunogens are mainly engineered instead of only selecting them from patients that developed the bNAbs. The pursuit of these approaches was triggered by realization that available Env immunogens did not engage the gl-precursors of bNAbs [101–103]. BG505 SOSIP.664 was no exception to this rule and did not interact with most gl-bNAbs, the exception being inferred versions of PG9/16, CH01 and 3BC315 [104]. The goal became to identify or design Env immunogens that do so. Focusing on gl-precursors of the VRC01-class of CD4bs bNAbs resulted in the design of the Env-based immunogens designated eOD-GT6/8 and 426c.TM4ΔV1-V3 [43,82,105]. These immunogens activate antibody responses in knock-in mice engineered to express the gl-precursors of VRC01 or 3BNC60 [43,44^▪^,^70^^▪▪^,^82^,^105^,106^▪^,^107^,^108^^▪▪^], and eOD-GT8 was also reported to prime VRC01-class precursors in human Ig loci transgenic mice [45^▪^]. Recently, we have engineered germline-targeting BG505 SOSIP trimers, termed BG505 SOSIP.v4.1-GT1 (GT for germline targeting) to engage gl-bNAbs *in vitro* and *in vivo*. BG505 SOSIP.v4.1-GT1 interacts efficiently with a subset of gl-precursors of NAbs targeting the trimer apex as well as the CD4bs [109]. Another BG505 SOSIP-based trimer has been engineered to engage gl-PGT121 [70^▪▪^]. After successful engagement of desirable gl-precursors, the respective B cells need to be shepherded with immunogens specifically designed to bridge the gap between germline and mature B cell responses until eventually the Env spike is recognized and neutralized [110]. Proof-of-concept studies in knock-in mouse models have shown that this is achievable. Thus, in one instance broad neutralization was achieved starting from the precursor of bNAb PGT121 [111^▪▪^]. In another example, broad neutralization was achieved starting with VRC01 precursor B cells, although in that case the neutralization was limited to viruses from which the 276 glycan was removed, showing that this glycan is probably one of the major roadblocks for inducing VRC01-class bNAbs [110].

In the “iterative design approach” Tier 2 NAb responses that are induced by native-like trimers can be used as a starting point. Thus, knowing that the autologous response against BG505 trimers is dominated by N241/N289 glycan hole responses, one can aim to broaden these responses by boosting with immunogens in which the these glycans are built back, to “teach” the hole-directed lineages to recognize the trimer in the presence of the respective glycans. This might relate to what happens in natural infection. For example, the VRC01-lineage was probably triggered by a virus that lacked the glycan at 276 then broadened when the glycan was reinstated [112,113]. Similar observations have been made for the PGT121-lineage and its adaptation to the presence of the 137 glycan [65]. In the BG505-infected patient, the 241 and 289 glycans emerge over time, although it is not clear whether that led to bNAb development in that patient. None of these approaches are mutually exclusive and in fact may be mutually supportive when used in a rational manner.

## CONCLUSIONS

The field of antibody-based vaccine design has rapidly come a very long way in the past 5 years with the discovery and characterization of a large spectrum of bNAbs and the development of stabilized trimers that closely mimic the functional Env spike on the virus. Initial immunization experiments with these next-generation trimers have already been carried out, and reveal that whilst autologous NAbs to Tier-2 virus Envs are achievable, the reproducible elicitation of high titer bNAbs is not. As described earlier, more sophisticated approaches to immunogen design, many based upon antigen stabilization, are underway, and immunization studies are following. Locking immunogens into a single stable conformation, as has been carried out for respiratory syncytial virus vaccine development [114,115], is likely to be important to facilitate efficient B cell recognition. The stability of the immunogen in the presence of adjuvant and in the complex *in vivo* milieu also needs to be taken into account. In addition to the approaches to immunogen design and regimens designed to trigger naïve BCRs proposed earlier, attention will also need to be given to the structure and immunogenicity of individual glycans that make up the Env glycan shield, as many of these are intrinsic components of bNAb epitopes. As we move along the path of designing a successful antibody-based vaccine to HIV-1 Env, we are learning a great deal about glycoprotein structural biology, how structure relates to immunogenicity, and how the adaptive immune system responds to a complex and moving target, all of which will inform vaccine approaches to other difficult pathogens.

## Acknowledgements

*None.*

### Financial support and sponsorship

*The work on native-like trimers in the Sanders and Sattentau labs is supported by the Bill & Melinda Gates Foundation (grants nos. OPP1111923 and OPP1132237 to R.W.S. and OPP1113647 to Q.J.S.); the National Institutes of Health (grant no. P01 AI110657 to R.W.S.); European Union's Horizon 2020 research and innovation programmes (grant nos. 681137 to Q.J.S. and R.W.S, and 681032 to Q.J.S.). R.W.S. is a recipient of a Vidi grant from the Netherlands Organization for Scientific Research (grant no. 917.11.314) and a Starting Investigator Grant from the European Research Council (grant no. ERC-StG-2011-280829-SHEV). M.M.-R. is a recipient of a fellowship from the Consejo Nacional de Ciencia y Tecnología (CONACyT) of Mexico. Q.J.S. is a Jenner Vaccine Institute Investigator and a James Martin Senior Fellow.*

### Conflicts of interest

*R.W.S. is listed as an inventor on patents related to native-like HIV trimers. There are no conflicts of interest for the remaining authors.*

## REFERENCES AND RECOMMENDED READING

Papers of particular interest, published within the annual period of review, have been highlighted as:

▪ of special interest
▪▪ of outstanding interest
